# Evaluation of some photosensitizers against the cotton leaf worm, *Spodoptera littoralis* (Lepidoptera: Noctuidae), in relation to spectral and thermal reflectance

**DOI:** 10.1038/s41598-024-81182-8

**Published:** 2024-12-23

**Authors:** M. S. Yones, Shireen A. M. Maamoun, Abd El Aziz A. Khidr, Mahmoud Sayed, Hend A. A. Al-Ashry, Radwa G. Attia

**Affiliations:** 1 National Authority for Remote Sensing and Space Sciences, Cairo, Egypt; 2 Entomology department, Faculty of Science, Ain Shams University, Cairo, Egypt; 3 Plant Protection Research Institute, Agricultural Research Center, Dokki-Giza, Egypt

**Keywords:** Noctuidae, Photosensitizing compounds, Hyperspectral reflectance, Thermal imaging, Biological techniques, Computational biology and bioinformatics

## Abstract

Photosensitizing compounds are eco-friendly promising organic dyes for managing insect pests without facing the risk of resistance. The photodynamic efficacy of four Photosensitizing compounds (rose Bengal, rhodamine B, methylene blue and methyl violet) was monitored against the third larval instar of *Spodoptera littoralis* (Boisduval), after exposure to sunlight. The LC_50_ values of the four compounds; rose Bengal, rhodamine B, methylene blue and methyl violet recorded 0.029 × 10^–5^, 0.24 × 10^–5^, 14.69 × 10^–5^ and 15.59 × 10^–5^ M, respectively. Therefore, rose Bengal was the most effective compound followed by rhodamine B, Methylene blue and methyl violet. Compared to similar previously documented photosensitizer compounds, these four tested compounds recorded higher mortality percentage. The effect of those compounds on the larval biochemical components was assessed by measuring larval spectral and thermal reflectance. Larvae fed on photosensitizing compounds showed distinct spectral reflectance patterns. Treated larvae had same reflectance manner; which were higher than that of control samples. Along the whole spectrum, the highest reflectance was after 60 min of exposure to sunlight in case of rose Bengal, rhodamine B and methylene blue. Methyl violet reflected sunlight mostly at zero time then the reflectance decreased after 30 min then increased again after 60 min. There was a noticeable absorption of IR wavelengths at ~ 1900, 1400, 1200 and 950 nm in all treated samples. Thermal imaging indicated abnormal larval warmth after treatment. Differences in reflectance were monitored immediately after feeding, confirming the speed and mode of action of those compounds.

## Introduction

The genus *Spodoptera* covers several significant polyphagous insects that are pests of many horticultural crops worldwide. From an agronomic point of view, the most important species of this genus are *Spodoptera exigua*, *Spodoptera frugiperda* (native to the tropical regions of the western hemisphere from the USA to Argentina), *Spodoptera littoralis* (Boisduval) (Europe and Africa) and *Spodoptera litura* (Fabricius) (Indo-Australian tropics). The cotton leaf worm, *Spodoptera littoralis* (Lepidoptera: Noctuidae), is a highly harmful pest to cotton crops in Egypt and has been recognized as one of the most destructive. It poses a significant threat to a variety of cultivated plants, resulting in considerable economic damages by consuming their vegetation as well as their reproductive structures^1^.

Developing an effective plan to manage the cotton leaf worm has been a key objective in agricultural entomology. However, utilizing chemical measures over extended periods of time has resulted in issues due to the presence of undesirable residues in food. The haphazard application of insecticides resulted in resistant pests to the majority of insecticides on the market. As a result, it is evident that to find alternative strategies for managing pests, that are safe to non-target organisms and to environment, are necessary and crucial. Furthermore, utilizing these tools in the field with minimal expenses is imperative. Photo-pesticides which are activated by sunlight, are an alternative to conventional chemical pesticides in this situation. Numerous laboratory and field studies on the corn rootworm^2^, the house fly^3^ and the Western Flower Thrips^4^, have investigated the use of photo-pesticides is promising for controlling various types of insects. Attia et al.^3^ evaluated the toxicity of rose Bengal, eosin yellow lactone and methylene blue against adult house fly, *Musca domestica.* LC_50_ values were 8 × 10^–5^, 7 × 10^–4^ and 1.050187 × 10–3 M, respectively. Results showed that rose Bengal was the most effective one, followed by eosin yellow lactone and methylene blue.

Photosensitizing compound are organic dyes which have pesticide effect. Rose Bengal and rhodamine B are photo-pesticides which belong to the class of organic compounds called xanthenes while methylene blue is a thiazine dye and methyl violet belongs to class triarylmethane dye. The photodynamic activity of such pesticides and their mechanism of action have been described by Heitz^5^. Toxicity of photosensitizers rely on the dye which causes cellular toxicity by acting as a catalyst for an oxygen molecule or reactive oxygen species (ROS) generation. The insect accumulates the photo-active substance which then induces toxicity at a cellular level upon visible light exposure. It causes harm to the outer layer (cuticle) and internal wall of the midgut, resulting in hindrance to feeding and eventually insect dies^6^. Additionally, they have a negative impact on the biochemical composition of insects^3,7^. In conclusion, photosensitizers have the ability to cause harm to cells through several mechanisms, including DNA fragmentation, oxidation of proteins, peroxidation of lipids, necrosis, and apoptosis. The process is continuous as photosensitizers can produce ROS continuously until they ultimately degraded. Certain photosensitizer classes can produce enough ROS which are lethal to insects^8^. These substances are only harmful to organisms that meet certain criteria, such as being small and transparent enough allowing light to enter through and subsequently activate the photosensitizers and they are safe for large and opaque living organisms^4^.

Several research studies have investigated the effect of different factors on the way plants reflect light. El Hoseny et al.^9^ explored and focused specifically on using reflectance profiling to distinguish between food products, with and without insect damage. Relatively few studies have determined the effect of stress on light reflectance patterns of organisms, either before or after treatment^10,11^. Thus, the objective is to determine how photosensitizing substances affect the reflectance of insect bodies.

Thermal remote sensing is a safe method for measuring the thermal properties of any interesting fact. The idea of thermal remote sensing rely on that invisible radiation from objects are translated as visible images known as thermal images^12^.

This work aimed to determine the toxicological effect of four photosensitizers (rose Bengal, rhodamine B, methylene blue and methyl violet) on the 3rd larval instar of cotton leaf worm. Then, investigating and tracking the photodynamic impact of the lc_50_ of the tested photosensitizers on the treated larvae by measuring their spectral and thermal reflectance.

## Materials and methods

### Rearing of insects

*Spodoptera littoralis* strain was acquired from the Cotton Leaf Worm Department, Plant Protection Research Institute, Agricultural Research Centre, Dokki, Giza, Egypt. A pure strain was raised, for more than six generations, under controlled circumstances without exposure to insecticides at 27 °C and 65–75% RH. Insects tested were fed daily on leaves of castor bean, according to El-Defrawi et al.^13^.

### Photosensitizing compounds used

i. Common name: Rose Bengal; (4,5,6,7-tetrachloro-2′,4′,5′,7′-tetraiodofluorescein). Trade name: Rosets; Chemical formula: C_20_ H_4_ Cl_4_ I_4_ O_5_; Molar mass: 973.67 g/mole; Quantal yield:076.
ii. Common name: Rhodamine B; 9-(2-Carboxyphenyl)-6-(diethylamino)-*N*,*N*-diethyl-3*H*-xanthen-3-iminium chloride. Trade name: Rhodamine 610; Chemical formula: C_28_ H_31_ Cl N_2_ O_3_; Molar mass: 479 g/mole; Quantal yield: 0.65
iii. Common name: Methylene blue; 3,7-bis (Dimethylamino)-phenothiazin-5-ium chloride. Trade name: Urolene blue; Chemical formula: C16H18N3SCl; Molar mass: 319.85 g/mole; Quantal yield: 0.52
iv. Common name: Methyl violet; Trade name: Crystal violet; Chemical formula: C25N3H30Cl; Molar mass: 408 g/mole; Quantal yield: 0.019.

### Bioassays

The four photosensitizers were serially diluted with water. Bioassay tests was conducted by dipping fresh leaves for 20 s in the serial concentrations of photosensitizing compounds. They were left at room temperature until complete drying. The 3rd larval instar was fed on the leaves in glass jars covered with muslin. Three replicates containing 20 larvae were done for each concentration of the tested compounds. Control (untreated larvae) were fed on leaves dipped in water only.

Larvae left for feeding an hour in dark (for measuring the effect of different photosensitizers on zero time), then they were taken out doors for direct exposure to sun light. Larvae were inspected every 15 min and observations were recorded till one and half hour after treatments.

### Spectral measurements

Spectral measurements were conducted in National Authority for Remote Sensing and Space Sciences (NARS), Nozha, Cairo, Egypt.

Hyperspectral spectroradiometer (ASD Field Spec 4 Hi-Res) of high resolution was utilized for monitoring the reflectance of the 3rd larval instar of S. *littoralis* in laboratory. Fifteen samples of about 15 ± 2 of 3rd larval instar treated with lc_50_ of rose Bengal, rhodamine B, methylene blue and methyl violet (three replicates for each treatment) and control. Reflectance was measured at zero time and at 2-time intervals post exposure to direct sunlight (after 30 and 60 min). Estimation of the larval samples was indicated through spectral range of 350 nm to 2500 nm [visible – Near-infrared (NIR)—Short Wave infrared (SWIR)].

### Thermal camera measurements

The information gathered by thermal remote sensing device (Testo 890 thermal camera) was captured on film, and recent temperature sensors of resolution of 0.1 °C. An illustration of relative radiant temperatures, with warmer values shown in lighter tones and cooler values in darker tones, for examination. False color images can be used to explain and examine how each pixel of an image relates to the surface temperature of an object.

### Statistical analysis

#### Bioassay statistics

Larval mortality percentages had been corrected using Abbott’s formula^14^. Mortality percentage statistics were calculated according to Finney^15^. The slope, LC_50_ and LC_90_ values were indicated. Sun’s toxicity index and potency levels were also estimated Sun^16^.

$${\text{Sun'}}{\text{s}}\;{\text{Toxicity}}\;{\text{index}} = \frac{{{\text{LC}}50{ }\;{\text{or }}\;{\text{LC}}90{ }\;{\text{of}}\;{\text{ the }}\;{\text{most }}\;{\text{toxic}}\;{\text{ compound}}}}{{{\text{LC}}50{ }\;{\text{or }}\;{\text{LC}}90{ }\;{\text{of }}\;{\text{the}}\;{\text{ tested }}\;{\text{other}}\;{\text{ compounds}}}}$$

$${\text{Potency }}\;{\text{levels}} = \frac{{{\text{LC}}50\;{\text{ or }}\;{\text{LC}}90{ }\;{\text{of }}\;{\text{the }}\;{\text{least }}\;{\text{toxic}}\;{\text{compound}}}}{{{\text{LC}}50{ }\;{\text{or}}\;{\text{ LC}}90{ }\;{\text{of }}\;{\text{the}}\;{\text{ tested }}\;{\text{other }}\;{\text{compounds}}}}$$

Analysis of variance was statistically estimated for indicating the significant differences between samples. Confidence level (5%) was used in all statistical analysis. CoStat^17^ software program was used for statistical tests.

## Results

### Toxicological studies

#### Mortality percentages, LC_50_ and LC_90_ levels

The toxicological effects of four different concentrations of the four photosensitizers against the 3rd larval instar of cotton leaf worm after 1 h and 15 min of sunlight exposure was evaluated.

As illustrated in Table 1, the LC_50_ values for the four compounds were arranged in a descending manner as follows: rose Bengal, rhodamine B, methylene blue and methyl violet recording LC_50_ values of 0.029 × 10^–5^, 0.24 × 10^–5^, 14.69 × 10^–5^ and 15.59 × 10^–5^ M; respectively. The corresponding LC_90_ were 2.59 × 10^–^54, 4.76 × 10^–5^, 53.84 × 10^–5^ and 64.78 × 10^–5^ M; respectively. Rose Bengal was the most toxic as it caused 35% mortality at concentration 0.01 × 10^–5^ M and 90% at concentration 1.5 × 10^–5^ M. In case of methyl violet, it caused the least mortality rates 24 and 81% at concentrations 4.8 × 10^–5^ and 24 × 10^–5^ M; respectively.

**Table 1:** Susceptibility status of the 3rd larval instar of *S. littoralis* to the four photosensitizers.

Photosensitizer	Conc. (M)	% Mortality	Slope	LC_50_(M)	LC_90_(M)
Rose Bengal	0.51 × 10^–7^	35.00	0.656	0.029 × 10^–5^	2.59 × 10^–5^
	0.51 × 10^–6^	50.00			
	0.51 × 10^–5^	70.00			
	0.77 × 10^–5^	90.00			
Rhodamine B	0.088 × 10^–5^	35.00	0.980	0.24 × 10^–5^	4.76 × 10^–5^
	0.175 × 10^–5^	40.00			
	3.500 × 10^–5^	85.00			
	4.00 × 10^–5^	90.00			
Methylene blue	6 × 10^–5^	24.00	2.296	14.69 × 10^–5^	53.84 × 10^–5^
	18 × 10^–5^	42.00			
	22 × 10^–5^	75.00			
	30 × 10^–5^	84.00			
Methyl violet	4.8 × 10^–5^	24.00	2.069	15.59 × 10^–5^	64.78 × 10^–5^
	14.4 × 10^–5^	39.00			
	18 × 10^–5^	72.00			
	2.4 × 10^–5^	81.00			

% mortality was determined after 4 h from exposure to sunlight.

### Estimation of toxicological parameter

To determine the effect of photosensitizers against the larvae, the toxicity index was calculated. For this study, rose Bengal was the standard compound and given 100 units as it was the most toxic compound used. Table 2 represent the toxicity indices of the compounds under investigation at both LC_50_ and LC_90_ levels. The three photosensitizing compounds, rhodamine B, methylene blue and methyl violet had toxicity indices of about 12.08 & 54.41, 0.20 & 4.81 and 0.19 & 4.00% as toxic as rose Bengal; at both LC_50_ and LC_90_; respectively.

**Table 2:** Toxicity index, slope, LC_90_/LC_50_ and potency levels of the four photosensitizers against the 3rd larval instar of *S. littoralis*.

Photosensitizer	Toxicity index based on		Slope	LC_90_/LC_50_	Potency levels	
	LC50	LC90			LC50	LC90
Rose Bengal	100	100	0.656	89.31	537.59	25.01
Rhodamine B	12.08	54.41	0.980	19.83	64.96	13.61
Methylene blue	0.20	4.81	2.296	3.67	1.06	1.20
Methyl violet	0.19	4.00	2.069	4.16	1.00	1.00

The slope values and LC_90_/LC_50_ ratios of photosensitizers treatments were calculated after 1 h and 15 min of larval exposure to sun light. The potency levels were estimated by dividing the LC_50_ or LC_90_ of methyl violet (least toxic) by the other photosensitizing products (Table 2). Methylene blue had the steepest toxicity line with slope of 2.296, whereas the flattest one was recorded for rose Bengal, with slope of 0.656. The slope value of the other compounds, rhodamine B and methyl violet were in the middle among the two previous compounds, with slope of 0.980 and 2.069; respectively.

Table 2 represent the potency levels for rose Bengal, rhodamine B and methylene blue. The potency levels of both LC_50_ and LC_90_ values were 537.59 & 25.01, 64.96 & 13.61 and 1.06 & 1.20 times as toxic as of methyl violet; respectively.

### Photodynamic effect of the four photosensitizers

The photodynamic effects of the four photosensitizers, with different concentrations, on larval mortality percentages were monitored at different intervals after sunlight exposure.

### Photodynamic effect of rose Bengal

First mortality recorded was at concentration of 0.01 × 10^−5^M and exposure to sunlight for 30 min. Whereas, mortality percentage recorded was 10%, and this percentage reached 35% mortality from 1 to 1 h and 15 min of sunlight exposure.

Treatment with 0.1 × 10^–5^ M, recorded 15% mortality after 30 min of exposure. After 45 min, larval mortality reached 30%. Then, percentage increased significantly to be 45 and 50% after an hour and hour and 15 min; respectively.

At concentration 1.00 × 10^–5^ M, mortality level was 35% after 0:30 h of exposure to sun light. Larval mortality attained 45, 60 and 70% after 0:45, 1:00 and 1:15 h; respectively.

Concentration of 1.5 × 10^–5^ M, recorded the greatest mortality rates which were 65% after half an hour, then the mortality levels were increased significantly to reach 90% after 1:15 h (Table 3).

**Table 3:** The photodynamic effect of different concentrations of rose Bengal on the 3rd instar larvae of* S. littoralis.*

Sunlight exposure periods (hrs.)	% Mortality at indicated concentrations expressed as mole			
	0.01 × 10^–5^	0.1 × 10^–5^	1 × 10^–5^	1.5 × 10^–5^
0:15	0.00^c^	0.00^d^	0.00^e^	0.00^c^
0:30	10^b^	15^c^	35^d^	65^b^
0:45	10^b^	30^b^	45^c^	85^a^
1:00	35^a^	45^a^	60^b^	85^a^
1:15	35^a^	50^a^	70^a^	90^a^
Control	0.0	0.0	0.0	0.0

Numbers with the different uppercase letters are significantly different in the same column.

### Photodynamic effect of rhodamine B

Rhodamine B lower concentration (0.09 × 10^–5^ M) showed mortality percentage 10% at 0:45 h of sunlight exposure. Percentage significantly increased to 15 and 35% after 1:00 and 1:15 h; respectively.

Concentration of 0.18 × 10^–5^ M caused 5% death after 0:30 h. At 0:45, 1:00 and 1:15 h, the percentages increased significantly to reach 20, 30 and 40%; respectively.

Higher concentration (3.5 × 10^–5^ M), gave about 10% mortality at 0:30 h, then reached 25 and 35% at 0:45 and 1:00 h. A significant increase in mortality was noticed 85% at 1:15 h from exposure.

The highest concentration of 4 × 10^–5^ M, caused 15% mortality at 0:30 h, then percentages increased significantly to record 90% larval mortality after 1:15 h (Table 4).

**Table 4:** The photodynamic effect of different concentrations of rhodamine B on the 3rd instar larvae of* S. littoralis.*

Sunlight exposure periods (hrs.)	% Mortality at indicated concentrations expressed as mole			
	0.09 × 10^–5^	0.18 × 10^–5^	3.5 × 10^–5^	4 × 10^–5^
0:15	0.00^d^	0.00^e^	0.00^e^	0.00^e^
0:30	0.00^d^	5^d^	10^d^	15^d^
0:45	10^c^	20^c^	25^c^	30^c^
1:00	15^b^	30^b^	35^b^	40^b^
1:15	35^a^	40^a^	85^a^	90^a^
Control	0.00	0.00	0.00	0.00

Numbers with the different uppercase letters are significantly different in the same column.

### Photodynamic effect of methylene blue

Table 5 showed methylene blue photo dynamic effect on cotton leaf worm larvae after sun light exposure. Concentration of 6 × 10^–5^ M of caused 5% larval mortality after 0:45 h. Mortality raised gradually to reach 24% after 1:15 h of exposure.

**Table 5:** The photodynamic effect of different concentrations of methylene blue on the 3rd instar larvae of* S. littoralis.*

Sunlight exposure periods (hrs.)	% Mortality at indicated concentrations expressed as mole			
	6 × 10^–5^	18 × 10^–5^	22.5 × 10^–5^	30 × 10^–5^
0:15	0.00	0.00^e^	5^d^	10^e^
0:30	0.00^d^	10^d^	25^c^	25^d^
0:45	5^c^	25^c^	50^b^	55^c^
1:00	15^b^	30^b^	55^b^	75^b^
1:15	24^a^	42^a^	75^a^	84^a^
Control	0.00	0.00	0.00	0.00

Numbers with the different uppercase letters are significantly different in the same column.

Larval mortality percentage reached 10% after 0:30 h, when treated with 18 × 10^–5^ M. The percentage increased significantly to 25% at 0:45 h. Then it reached 30% after an hour. The mortality level reached 42% after 1:15 h of exposure.

Higher concentration of 22.5 × 10^–5^ M, attained mortality of about 5% at 0:15 h. Mortality level increased significantly to 25% at 0:30 h. After that a gradual increase in mortality was recorded to be 50, 55 and 75% after 0:45, 1:00 and 1:15 h; respectively.

Concentration of 30 × 10^–5^ M, at first caused mortality of 10% after 0:15 h of exposure, then mortality increased to 25% at 0:30 h. Gradual increase in mortality percentages was noticed, to reach its maximum (84%) after 1:15 h.

### Photodynamic effect of methyl violet

Results illustrated in Table 6 showed that concentration of 4.8 × 10^–5^ M of methyl violet caused 6% mortality percentage at 0:45 h from sunlight exposure. Percentage significantly increased to reach 24% mortality after 1:15 h.

**Table 6:** The photodynamic effect of different concentrations of methyl violet on the 3rd instar larvae of* S. littoralis.*

Sunlight exposure periods (hrs.)	% Mortality at indicated concentrations expressed as mole			
	4.8 × 10^–5^	14.4 × 10^–5^	18 × 10^–5^	24 × 10^–5^
0:15	0.00^d^	0.00^d^	10^e^	15^e^
0:30	0.00^d^	10^c^	25^d^	30^d^
0:45	6^c^	12^c^	42^c^	57^c^
1:00	18^b^	21^b^	60^b^	69^b^
1:15	24^a^	39^a^	72^a^	81^a^
Control	0.00	0.00	0.00	0.00

Numbers with the different uppercase letters are significantly different in the same column.

For concentration 14.4 × 10^–5^ M, mortality recorded was 10% after half 1 h of exposer. Mortality level slightly increased to attend 12% after 0:45 h. Then percentage increased to 21% after 1 h. The highest mortality percentage exhibited 39% after 1:15 h of exposure.

Concerning to 18 × 10^–5^ M of methyl violet concentration, mortality was 10% at 0:15 h. Mortality rate raised significantly to record 25, 42, 60 and 72% at 0:30, 0:45, 1:00 and 1:15 h; respectively.

Treatment of 3rd instar larvae with the highest concentration 24 × 10^–5^ M of methyl violet, caused the earliest 15% larval mortality after 0:15 h, the percentage increased to 30% at 0:30 h after sun exposure. Significant increase in 3rd larval mortality treated with methyl violet to reach 81% after 1:15 h.

### Median lethal time for the different concentrations of the four photosensitizing compounds

**Fig. 1:**
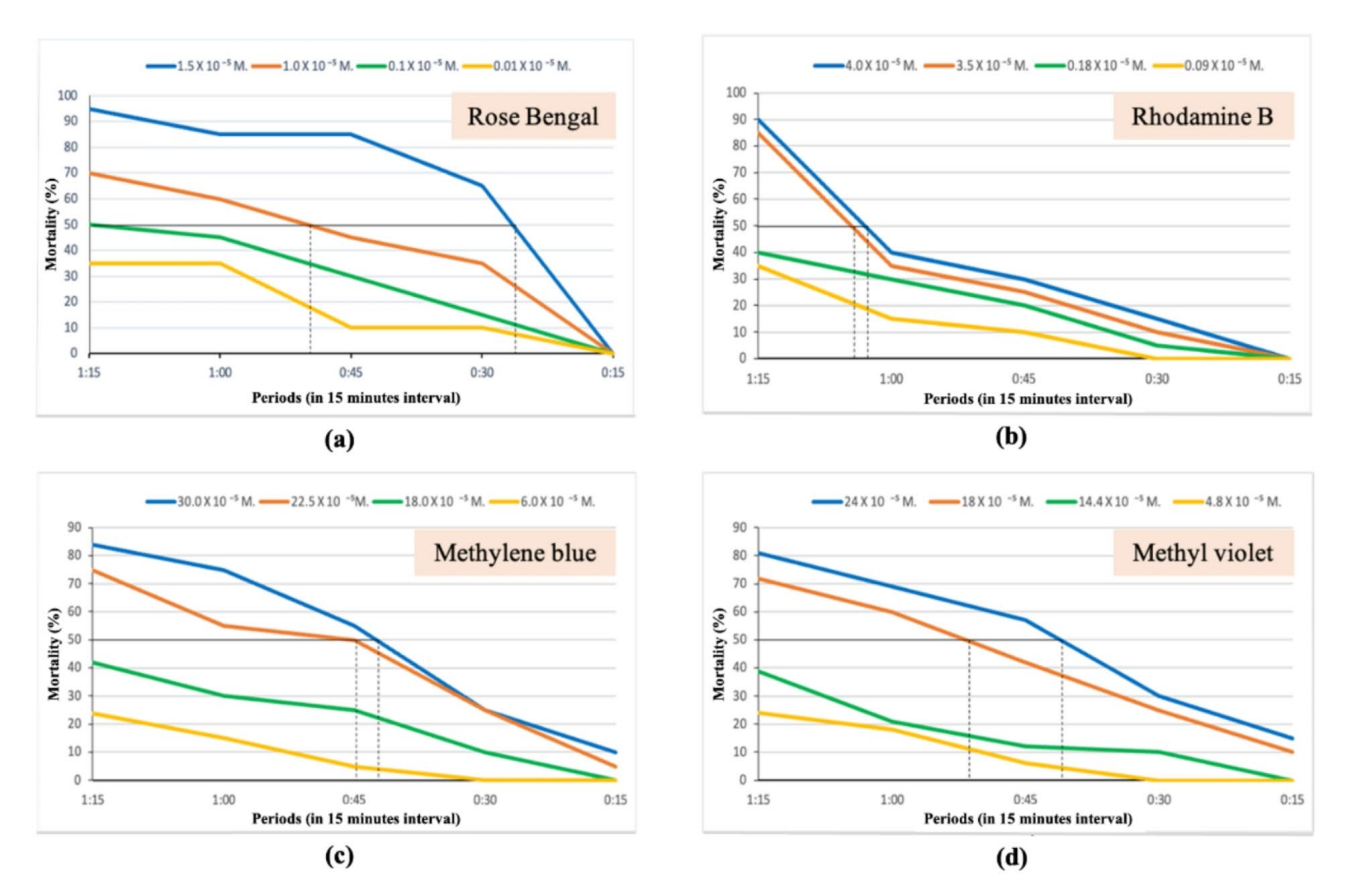
Effect of different concentrations of photosensitizing compounds on the mortality percentages of *S. littoralis* 3rd instar larvae exposed to sun light for different time intervals; (**a**) Rose Bengal, (**b**) Rhodamine B, (**c**) Methylene blue and (**d**) Methyl violet.

A median lethal time (LT_50_) based comparison between different photosensitizers is illustrated in Table 7. The LT_50_ of rose Bengal for the concentration of 0.01 × 10^–5^ M is greater than 1:15 h and at concentrations of 0.1 × 10^–5^, 1.0 × 10^–5^ and 1.5 × 10^–5^ M were 1:15, 0:55 and 0:25 h; respectively (Fig. 1a).

**Table 7:** Median lethal time (LT_50_) of the four photosensitizers against the 3rd larval instar of *S. littoralis* exposed to sun light for different time intervals.

Photosensitizing compounds	Conc. (M)	LT_50_ (hrs.)
Rose Bengal	0.01 × 10^–5^	> 1:15
	0.1 × 10^–5^	1:15
	1.0 × 10^–5^	0:55
	1.5 × 10^–5^	0:25
Rhodamine B	0.09 × 10^–5^	> 1:15
	0.18 × 10^–5^	> 1:15
	3.5 × 10^–5^	1:10
	4 × 10^–5^	1:05
Methylene blue	6 × 10^–5^	> `1:15
	18 × 10^–5^	> 1:15
	22.5 × 10^–5^	0:45
	30 × 10^–5^	0:42
Methyl violet	4.8 × 10^–5^	> 1:15
	14.4 × 10^–5^	> 1:15
	18 × 10^–5^	0:52
	24 × 10^–5^	0:40

LT_50_ values of Rhodamine B for lower concentrations of 0.09 × 10^–5^ and 0.18 × 10^–5^ M were more than 1:15 h; however, the two higher concentrations 3.5 × 10^–5^ and 4 × 10^–5^ M, the LT_50_ recorded were 1:10 and 1:05 h; respectively (Fig. 1b).

Methylene blue, LT_50_ at lower concentrations of 6 × 10^–5^ and 18 × 10^–5^ M were > 1:15 h. But higher concentration of 22.5 × 10^–5^ M, recorded 0:45 h. Highest concentration of 30 × 10^–5^ M attained 0:42 h (Fig. 1c).

For the photosensitizing methyl violet, similarity of LT_50_ values were noticed as mentioned to rhodamine B and methylene blue compounds, values of LT_50_ at concentrations 4.8 × 10^–5^ and 14.4 × 10^–5^ M were greater than 1:15 h. While concentrations of 18 × 10^–5^ and 24 × 10^–5^ M, recorded 0:52 and 0:40 h; respectively (Fig. 1d).

### Spectral measurements

**Fig. 2:**
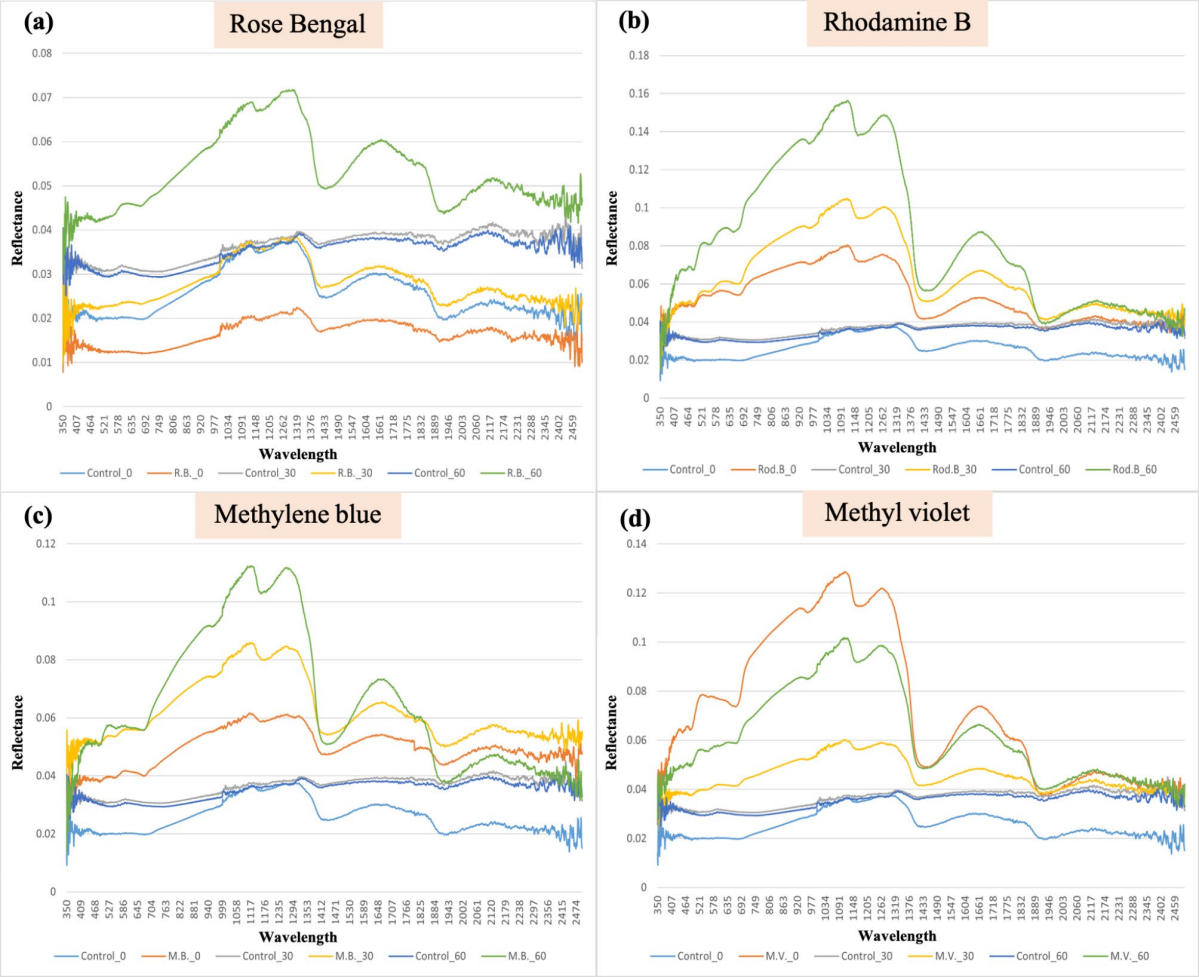
The Spectral Reflectance Patterns for the 3rd instar larvae of *S. littoralis* treated with photosensitizers; (**a**) Rose Bengal, (**b**) Rhodamine B (**c**), Methylene blue and (**d**) Methyl violet, compared with the control after sunlight exposure for different intervals of time.

The spectral reflectance patterns for the 3rd instar larvae of *S. littoralis* treated with photosensitizing compounds in relation to the untreated larvae was investigated. All reflectance patterns were of the same manner; as though the reflectance of larvae that fed on photosensitizing compounds was greater than that of all other control samples. The reflectance of larvae that fed on photosensitizing compounds after 60-min exposure to sunlight is higher than the reflectance after 30-min in rose Bengal, rhodamine B and methylene blue all over the whole spectrum (Fig. 2a–c). While methyl violet appeared to reflect sunlight mostly at zero time then the reflectance decreased significantly after 30 min then increased again after 60 min (Fig. 2d). Larvae treated with rose Bengal also showed the lowest reflection manner on the whole spectral bands.

The increase in reflectance of different spectral bands is obvious in Green, Red, NIR, and SWIR2 bands for rose Bengal and methylene blue. In case of rhodamine B and methyl violet the increase appears clearly in Red, NIR, and SNIR1 bands (Fig. 2).

### Thermal imaging measurements

**Fig. 3:**
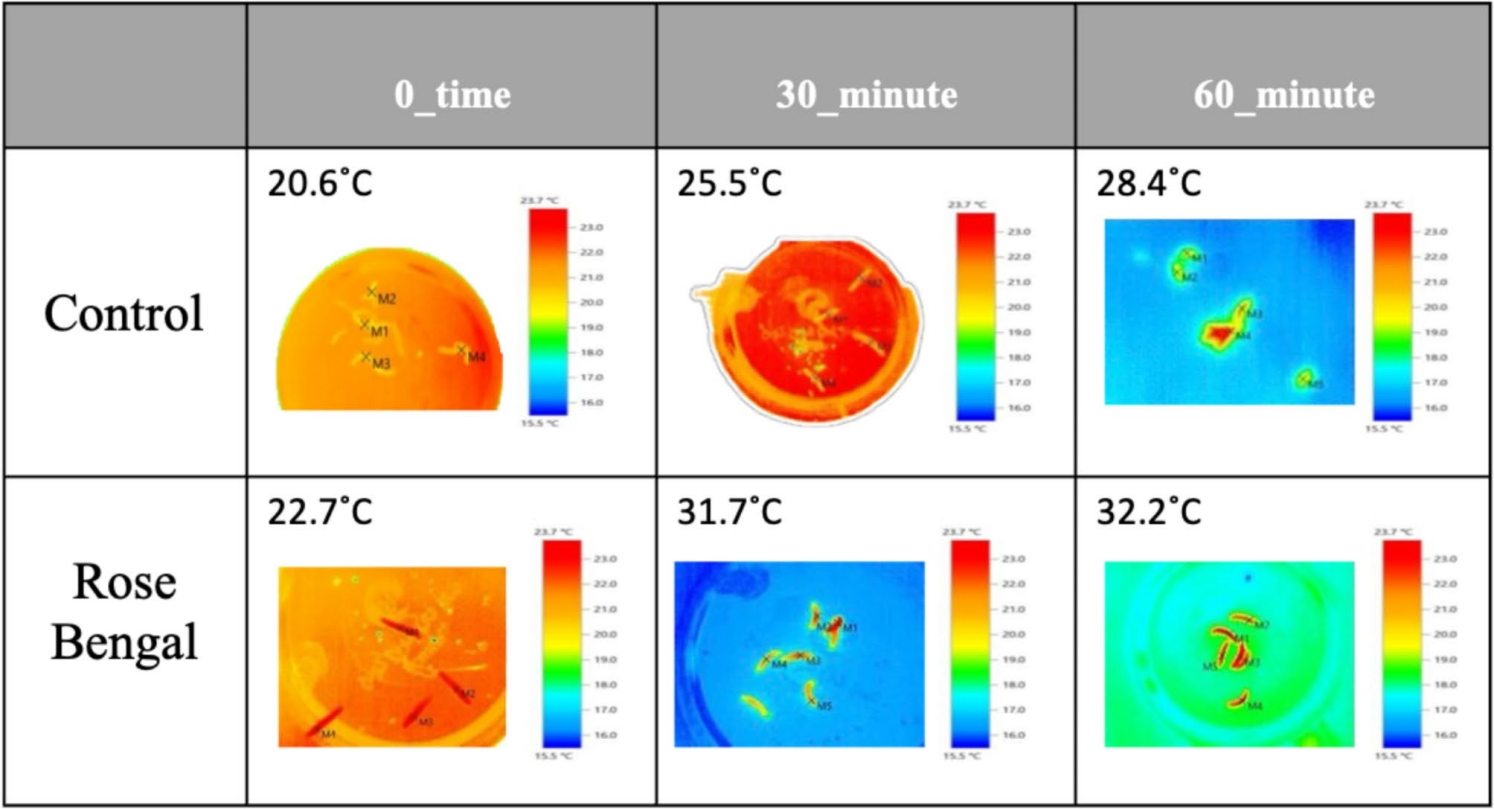
Thermal imaging of the 3rd instar larvae of *S. littoralis* treated with Rose Bengal at different time intervals of exposure to sunlight, compared with the control.

**Fig. 4:**
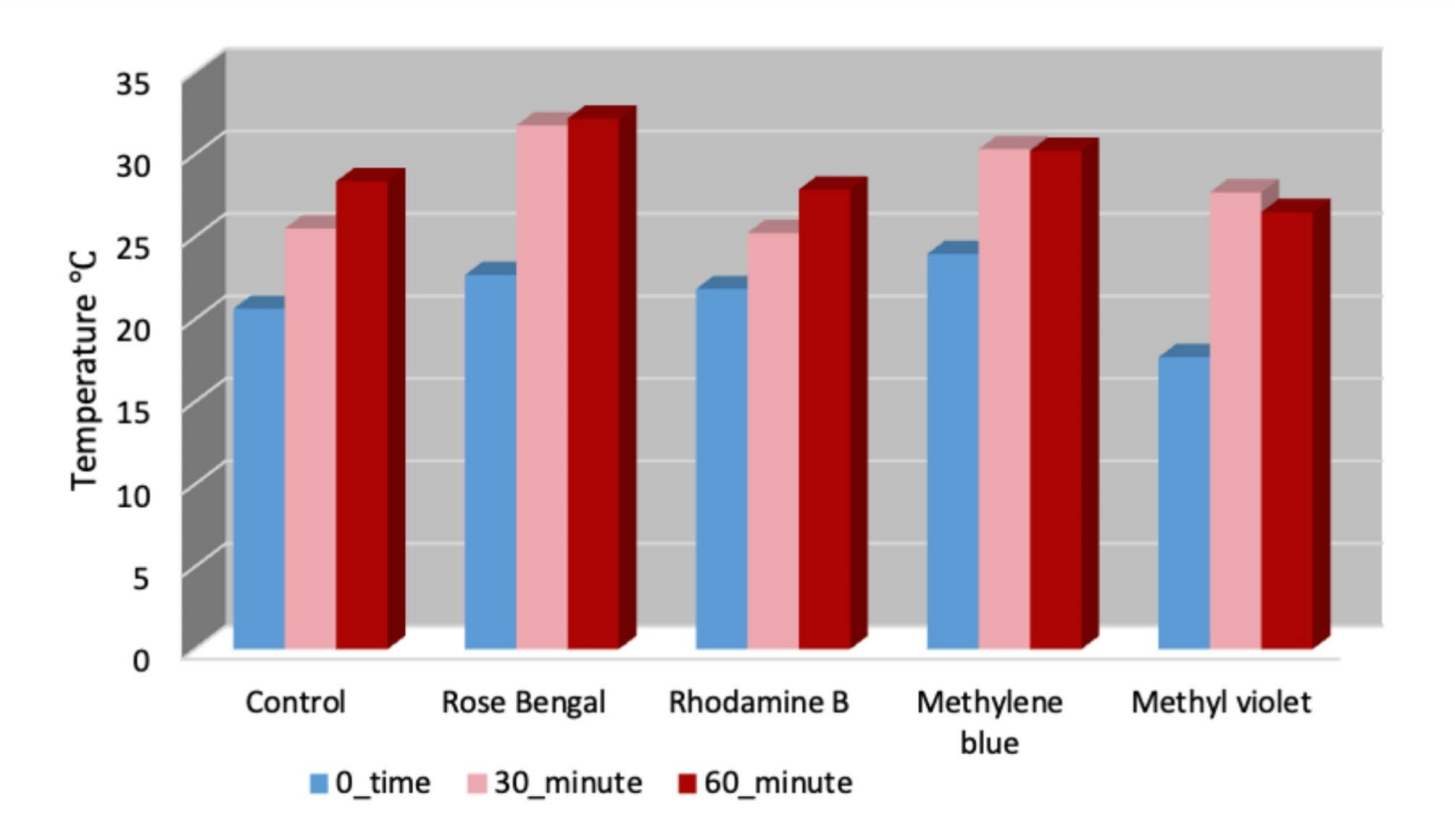
A thermal comparison chart for different photosensitizers; Rose Bengal, Rhodamine B, Methylene blue and Methyl violet, at different time intervals of exposure to sunlight, compared with the control.

Thermal imaging of the cotton leafworm larvae indicated abnormal warmth after treatment. The maximum temperature differences (MTD) between healthy cotton leafworms and others with photosensitizing compounds were investigated. Temperature of treated larvae was found to be of 22.6 °C, 21.8 °C, and 23.9 °C, at zero time (without sun light exposure), with temperature difference from untreated larvae about 2 °C, 1.2 °C and 3.3 °C for rose Bengal, rhodamine B and methylene blue; respectively. For Methyl violet, the temperature decreased significantly ~ 3 °C at zero time but temperature increased again after 30 and 60 min of sunlight exposure. Thermal imaging showed the temperature distribution on the larval surface after 30 and 60 min of sun light exposure (Fig. 3). It was noticed that the temperature of treated cotton leafworm was 2.2 to 6.2 °C higher than that of untreated larvae, except rhodamine B which pretend to be the same as control after 30 min of sunlight exposure. The range after 60 min of exposure increased to be about 2 °C and 4 °C in case of methylene blue and rose Bengal; respectively, which is considered a lower temperature difference than that recorded after 30 min of sunlight exposure in comparison with control after 30- and 60-min. Rhodamine B and methyl violet decreased the temperature of treated larvae about 0.5 °C and 1.9 °C than control; respectively (Fig. 4).

## Discussion

It is important to realize that conventional insecticides have a crucial role in all control programs of cotton leaf worms. However, the subsequent development of resistance to several pesticides currently in use, suggested that further work is required to identify efficient alternative control strategies. Due to their unique mode of action, photosensitizers may be able to control insect pests effectively without the risk of resistance build-up. Photosensitizers can do toxicity against different insects by different effects such as effect on biochemical contents, ultrastructure of the insect, change the genomic structure of DNA and sequences, and were not affected by the detoxifying of enzymes activity^3,5^. Also, as a physical process, photosensitizers has attracted attention because no microorganism resistance is expected to be developed during the photoinactivation treatment, and it can be applied to microorganism inactivation regardless of the multidrug resistance as well^18^.

Sustainable, environmentally friendly control stuffs are desperately needed to combat insect infestations and diseases all over the world. Several investigations photosensitizer could replace conventional chemical insecticides in the control of pests^3,4^.

According to Heitz^5^, Attia^3^and Shiao et al.^8^, the generation of singlet oxygen, which has toxicological and metabolic consequences on insects, is a key component of the light-dependent process of xanthene chemicals. The current investigation is a trial that contribute in such studies.

Four photosensitizers were concerned for their toxicity against cotton leaf worm larvae. The treated larvae displayed various levels of susceptibility to different photosensitizers used. LC_50_ values were between 0.029 × 10^–5^ and 15.59 × 10^–5^ M. This supports earlier findings of similar studies with different photosensitizers in both *vitro* and field applications^3,19^. The LC_50_ values of the four tested compounds; rose Bengal, rhodamine B, methylene blue and methyl violet recorded 0.029 × 10^–5^, 0.24 × 10^–5^, 14.69 × 10^–5^ and 15.59 × 10^–5^ M, respectively. Similar photosensitizers recorded lower toxicity as compared to our results of the four tested compounds. Respicio and Heitz^20^ used rhodamine 6G to control house fly adults and the LC_50_ was estimated as 0.87 × 10^–3^ M after 24 h of feeding period. Berni et al.^21^ tried to control the immature stages of the Mediterranean fruit fly, *Ceratitis capitata* by using phloxine B. They calculated the value of LC_50_ that was 0.11 × 10^–1^ M in completely dark conditions.

Obtained results showed that rose Bengal was the most effective one, followed by rhodamine B. Several studies had noticed the same outcome on other insect species^3,22^. Rose Bengal Photodynamic processes, have been concerned to be hundred times more active than the widely used chlorpyrifos pesticide and efficient against insecticide-resistant mosquitoes^5^. However, methylene blue and methyl violet had relatively low toxicity against *S. littoralis* larvae; this compatible with result obtained on tomato leaf miner^23^, and house fly adults^3^. On the other way around, Graham et al.^24^ mentioned that methylene blue was extremely poisonous to yellow mealworm larvae. The variations might be according to different susceptibility of insect species under investigation. Also, El-Ghobary et al.^7^ revealed that the potency of the toxicity index of methylene blue and rose Bengal are relatively the same against the cotton leafworm. On contrary, in the present study methylene blue and methyl violet had the lowest toxicity levels and rose Bengal was highly toxic. Mangan and Moreno^25^ illustrated that feeding intensity and the amount of ingestion by the insect species affect the photosensitizers toxicity and it depends on time of exposure to sunlight.

Referring to the composition of the photosensitizers, rose Bengal has the majority of halogen atoms by containing, 4 iodine and 4 chlorine atoms; while rhodamine B, methylene blue and methyl violet has only one chlorine atom. Therefore, halogen atoms enhance and amplify the yield of compound toxicity^3,26^. Photosensitizers toxicity could also be explained by their singlet oxygen quantum yield. There were correlations observed between singlet oxygen quantum yield (the number of molecules of ^1^O2 generated for each photon absorbed by a photosensitizer), and the photodynamic action effectiveness^5,24,27,28^, where the singlet oxygen quantum yield of Rose Bengal, Rhodamine B, Methylene blue and Methyl violet are 0.76, 0.65, 0.52 and 0.019, respectively.

The absorption and reflection of UV–Vis spectroscopy is a suitable technique for determining how light can affect dyes^29^. Photosensitizing compounds are dyes that accumulates in the insect tissues and enhance some photo-chemical reactions that are lethal to insects^22^. Absorption of light, whether artificial or natural, at specific wavelengths is required for photosensitizers to act properly and improve their efficacy as pesticides^30^.

Cotton leaf worm 3rd instar larvae are relatively small and partially translucent, so they allow light to penetrate through their bodies and consequently activate photosensitizers. This study focused on tracking the photodynamic absorption and reflection of different, wide range, spectral bands in and from treated larvae with photosensitizing compounds.

Because the radiometric energy in the 400–900 nm spectral regions penetrate many millimeters deep^31^. It appears plausible to expect that hyperspectral measuring of insect bodies, as employed in the present study, can be applied without causing harm to insects to identify and to monitor internal physiological and biochemical reactions (including stress response to killing agents).

As a result, the findings seem to confirm the idea that health status of insects can be determined non-destructively by analyzing their body reflectance patterns. This result agreed with finding of Nansen et al.^10^.

Hyperspectral results revealed that, along the whole spectrum, the reflectance of larvae that fed on photosensitizing compounds after 60-min exposure to sunlight is higher than the reflectance after 30-min. Difference in reflectance through different treatments after different time intervals may be explicated by light falls on living tissues. When light falls on insect body, it is exposed to different scattering patterns as a result of the inhomogeneous structure and components of tissues. Light also is exposed to various absorption forms by different biological molecules, like hemoglobin, melanin, and water^32^. It is supposed that the tissue’s absorption and reflection properties differ at various stages of infection^33^. Consequently, hyperspectral imaging could estimate a quantitative diagnosis from the transmitted and reflected light^33^.

Tissues contain molecules that can absorb light which are called chromophores. Chromophores such as melanin and blood components are absorbers for visible wavelengths, while proteins and amino acids absorb Ultra violet (UV) and water absorb Infra-red (IR) wavelengths^34^. Treatment with photosensitizing compounds generate ROS that are harmful to cells via DNA damage, protein oxidation, lipid peroxidation, necrosis, and inducing apoptosis^8^. As a result of these high rates of metabolic processes, water molecules were produced in excess manner which caused the noticed absorption of IR wavelengths at ~ 1900, 1400, 1200 and 950 nm (Fig. 2). As, water absorbs light bands around 1950, 1450, 1200 nm and 970 nm which fell within the Near infra-red range. Reflectance of visible light throughout the larval body may be due to changes in blood and skin colors. This indicates the rapid translocation of the photosensitizing compounds with their different colors to different body parts from zero time of treatment until an hour later. As a conclusion, the dyes rapidly reached all insect body parts and negatively affect the insect metabolism. That was indicated by the presence of the excess water measured by the spectroradiometer.

Thermal imaging indicated abnormal warmth of larvae after treatment. It indicated the distribution of temperature on larval surface after 30 and 60 min of sunlight exposure. At first after 30 min of sunlight exposure temperature significantly increased but this rate of increase decreased again after 60 min. These decreases in temperature may be due to increase in water production inside the larvae as a result of sudden increase in metabolic rates^8^. It was noticed that after immediate eating photosensitizing compounds there was an increase in temperature in most compounds, but the increase was mostly recorded after 30 min of sunlight exposure. Results revealed that photosensitizers may have an effect before sunlight exposure but this effect highly increased after exposure. As, results indicated that difference in temperature between untreated and treated cotton leafworm was substantial. Thermal remote sensing is a promising technique that could be used to differentiate between infected and healthy tissues with high precision.

The pest control industry became aware that IR thermography is useful in recognizing pest infestations by investigating the signs of latent moisture inside structures. Thermal imaging for determining thermal changes caused by insect infestation, data validation, and problems associated checking process were represented by Grossman^35^.

According to previous speculations, thermal imaging now can detect early physiological responses of cotton leafworm to water stress. In order to test this assumption, the temperature was taken in consideration. Martynenko et al.^36^ by the same concept, used thermal imaging to differentiate between healthy and diseased soybean plants under the same conditions.

## Conclusions

In recent years, food security and climate change are becoming increasingly important issues. So, there is a persistent need to switch towards ecologically conscious sustainable agriculture and food production. Resistance to insecticides has grown in several common insect pests. One of the most pervasive and harmful pests to cotton and other cultivated plants is the cotton leaf worm. Alternatives like photosensitizers may be able to efficiently reduce pest infestations, especially rose Bengal and rhodamine b, without the risk of resistance developing. This study proved that, photosensitizers do really work and have toxic effect on cotton leaf worm after exposure to sun light. Their effect is rapid and cause physiological changes inside the insect body which could be noticed clearly by hyperspectral measurements and thermal imaging.

## Data Availability

All data generated or analyzed during this study are included in this published article.
